# Sexually Transmitted Infection Rates and Closure of Family Planning Clinics Because of Abortion Restrictions in Iowa

**DOI:** 10.1001/jamanetworkopen.2022.39063

**Published:** 2022-10-14

**Authors:** Megan Srinivas, Ngai Sze Wong, Robin Wallace, Michael Emch, Weiming Tang, Eileen J. Yang, Joseph D. Tucker

**Affiliations:** 1 Institute for Global Health and Infectious Diseases, University of North Carolina at Chapel Hill, Chapel Hill; 2 Jockey Club School of Public Health and Primary Care, Chinese University of Hong Kong, Shatin, Hong Kong; 3 Stanley Ho Centre for Emerging Infectious Diseases, Chinese University of Hong Kong, Shatin, Hong Kong; 4 Student Health Service, University of North Carolina at Chapel Hill, Chapel Hill; 5 Department of Geography, University of North Carolina at Chapel Hill, Chapel Hill; 6 Department of Biostatistics, University of Michigan, Ann Arbor; 7 Clinical Research Department, Faculty of Infectious and Tropical Diseases, London School of Hygiene and Tropical Medicine, London, United Kingdom

## Abstract

This cohort study assesses rates of sexually transmitted infections in Iowa counties before and after closure of family planning health centers and compared with national rates.

## Introduction

Dismantling abortion services may have unintended consequences for essential primary care services,^1^ especially screening for and treating sexual transmitted infections (STIs). One way to restrict abortion services in the US has been through closing publicly funded family planning health centers (FPHCs), which play a key role in providing abortions and STI services in the US.^2^ Among people in the US who use FPHCs, a substantial number use these centers as their only source of health care.^3^ In the US, 18 states have abortion-related defunding policies restricting Medicaid reimbursements or Title X funds.^4^ Many FPHCs have closed because they provided abortions. In May 2017, Iowa state legislation restricted funding to FPHCs that provided abortion and STI services in 4 counties (Des Moines, Lee, Scott, and Woodbury). Previous studies have demonstrated the association of closing US FPHCs with unintended pregnancies, but to our knowledge, none have focused on STIs. This study aims to analyze the change of STI rates before and after FPHC closures.

## Methods

This cohort study was approved by the University of North Carolina Internal Review Board. Informed consent was waived because this was determined to be nonhuman participants research and only used deidentified data.

We collected data from the Centers for Disease Control and Prevention AtlasPlus Database for annual reported gonorrhea and chlamydia diagnoses from 2010 to 2019 in all Iowa counties. Differences in chlamydia and gonorrhea case rates per 100 000 persons in 2016 (preclosure period) and 2018 (postclosure period) were compared between Iowa’s counties with and without clinic closures using a Mann-Whitney *U* test. To account for time trends, Poisson regression for rates in 2000 to 2018 (exclusion of 2017 data), using the number of cases as the outcome of interest and county-level population as the offset, was conducted to determine if clinic closures had any association with gonorrhea and chlamydia cases in 2018. We also did a sensitivity analysis using a difference-in-difference regression and another restricted to counties with a federally qualified health center. *P* values were 2-tailed, and statistical significance was set at *P* < .05. Analyses were conducted using R version 4.2.1 (R Project for Statistical Computing). Data were analyzed from January 15 through September 4, 2022.

## Results

**Figure 1.**
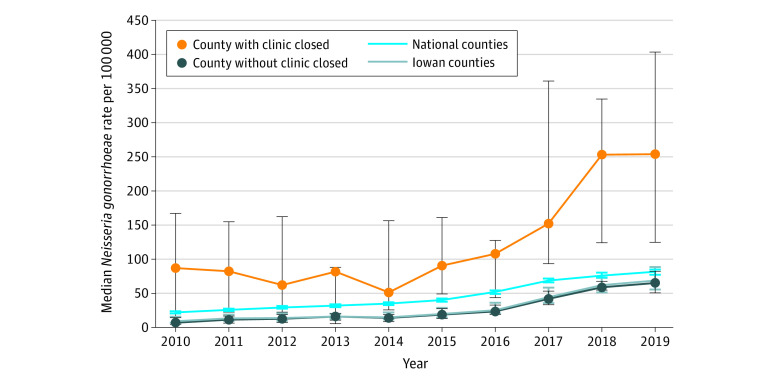
Clinic-Reported Gonorrhea Cases per 100 000 Population From 2010 to 2019 Data from the US Centers for Disease Control and Prevention AtlasPlus Database. The dots indicate medians; whiskers, 95% CIs.

**Figure 2.**
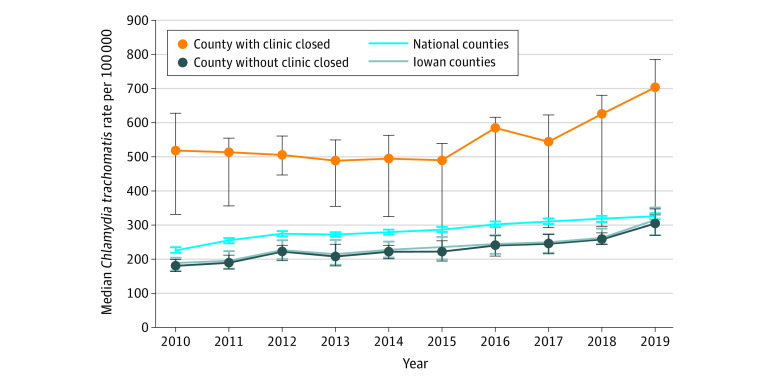
Clinic-Reported Chlamydia Cases per 100 000 Population From 2010 to 2019 Data from the US Centers for Disease Control and Prevention AtlasPlus Database. The dots indicate medians; whiskers, 95% CIs.

A total of 27 595 gonorrhea and 125 068 chlamydia cases were reported in 2010 to 2019. Statewide gonorrhea burden significantly increased from 2016, the year immediately prior to clinic closure (83 cases per 100 000 population) to 2018, the year following clinic closure (153.7 cases per 100 000 population) (Figure 1). Statewide chlamydia cases also increased from 2016 (414.6 cases per 100 000 population) to 2018 (466.3 cases per 100 000) (Figure 2). Iowa’s counties with clinic closures had a significantly larger increase in gonorrhea case rates (*U* = 37; *P* = .003) between 2016 and 2018 compared with counties without clinic closures. In Poisson regression, Iowa residents were approximately twice as likely to have a reported gonorrhea infection after closure (2018) compared with the period prior to closure (2000-2016) (rate ratio, 1.99; 95% CI, 1.67-2.37). The difference between 2016 and 2018 for chlamydia rate was not significant (*U* = 149.5; *P* = .49). The difference-in-difference regression showed similar results. When we restricted the analysis to counties with a federally qualified health center, there was still a significant difference between the gonorrhea rates between counties with clinic closure and those without closures (*U* = 18; P = .03).

## Discussion

The findings of this cohort study suggest that restricting abortion services by closing FPHCs was associated with delivery of essential STI services. Despite having fewer clinics reporting STIs during the later period, there were substantial increases in gonorrhea and smaller increases in chlamydia, particularly in areas with clinic closures. This study is limited by a small number of counties with closures, limited STI data, and variable integration between abortion and STI services. Nonetheless, our study extends the literature on how abortion restrictions may impact essential primary care services in the US.^5^ In the wake of *Dobbs v Jackson*, approximately 36 million people will lose access to abortion services.^6^ Ensuring access to essential STI services amidst this disruption is important.
